# Enucleation of the Eye in Sympathetic Ophthalmia

**Published:** 1869-10

**Authors:** Eugene Smith

**Affiliations:** Detroit


ART. III.—*Enucleation of the Eye in Sympathetic Ophthalmia.* By EUGENE SMITH, M. D., Detroit.

Much has already been written concerning the removal of the globe in Sympathetic Ophthalmia from traumatic causes, the propriety of which is well established; but very little has been said about Enucleation in Sympathetic Ophthalmia from other causes. Many have become totally blind, whose sight might have been saved had they fallen into the hands of a physician who had studied the subject sufficiently to estimate the danger, thereby knowing how to successfully combat the disease. We cannot expect physicians in general practice to devote enough attention to diseases of the eye to become experts in their diagnosis and treatment; but we have a right to expect, and we sincerely hope that they will inform themselves well enough to treat, or to *leave alone*, the most dangerous disease.

When an eye from disease has undergone abnormal changes, its fellow-eye is particularly liable to be attacked by Sympathetic Ophthalmia, which is an alarming and intractable disease. Premonitory symptoms very frequently precede the outbreak. One of its earliest symptoms is muscæ volitantes, followed by great sensitiveness and intolerance of light, straining of the accommodative apparatus, severe ciliary neurosis, feeling of pressure and tension, frequent attacks of pain radiating over the head, and by episcleral congestion, with occasional attack of iritis, which often produces posterior synechiæ. The sympathetic excitation of the nerves, however, does not always lead to exudative iritis. In some cases the disease is only indicated, for a long time, by severe photophobia and incapability for use; or by photophobia, with periodical darkening, for a short time, of the visual field. Cases, also, occur in which a rapidly increasing amblyopia, with development of a glaucomatous excavation of the optic nerve, arises. The last named condition is most often seen in old persons, and is always connected with a decided increase in the hardness of the globe.

The indications for treatment are, the removal of the conditions that excite the inflammatory process, as well as the direct removal of the inflammation, and the accompanying disorders of the circulation, and of the nervous system.

Where the abnormal condition of the opposite eye points to that as the exciting cause, enucleation is not only justified but necessary, and in fact the only *sure* method of arresting the sympathetic disease, and should be practised at an early stage of the disease, otherwise its beneficial effects will not be obtained. Time should not be wasted in trifling experiments, which may be destructive.

The propriety of not undertaking the enucleation, except during marked symptoms of inflammation, has recently received much attention, because the operation has, in some cases, been seen to be of little or no use performed when the inflammation was at its height; it is, however, very unsafe, and even dangerous in the majority of cases, to wait for a marked omission. Absolute rest of the sympathetically affected eye should be strictly enjoined, with the frequent application of atropine in solution, or belladonna lotion.

The following case illustrates the benefit of enucleation performed early. Mr. M., aged 18, consulted me last March with regard to severe inflammation of the left eye, and symptoms of sympathetic participation in the other eye. The left eye was in a state of sclero-choroidal staphyloma, which first made its appearance six months previous, about which time he was first attacked with inflammation, the exact cause of which he did not know. He was treated by the family physician for three weeks without getting better, when his father brought him to Detroit; and he was placed in the hands of a quack for treatment, who, it seems, succeeded in abating the inflammatory symptoms—not, however, until the globe had become partially staphylomatous. He returned home, and was for three months free from suffering, when, without any apparent cause, the eye again became inflamed, but was easily controlled. After this second attack, the exacerbations were frequent and easily excited. A few days before he called on me, he was struck on the eye with a stick, inflammation immediately set in, the staphyloma increased rapidly, and the well eye showed sympathetic symptoms, such as dimness of vision, photophobia, pain, &c. As those symptoms increased, he thought it time to consult an oculist. The right eye showed such marked symptoms of Sympathetic Ophthalmia, I advised enucleation of the globe of the left eye as the only hope for saving the other. Having already suffered severely, he gladly assented to the operation, which was performed in the usual manner. The wound healed kindly, the symptoms of irritation in the healthy eye soon disappeared, and the patient is now able to wear an artificial eye with comfort.

Ophthalmology has received too little attention from physicians in general practice; and we hope the foregoing case and remarks will be of sufficient interest to impress the importance of the subject upon the minds of all under whose notice they may fall.

